# A minimally invasive neurostimulation method for controlling abnormal synchronisation in the neuronal activity

**DOI:** 10.1371/journal.pcbi.1006296

**Published:** 2018-07-19

**Authors:** Malbor Asllani, Paul Expert, Timoteo Carletti

**Affiliations:** 1 naXys, Namur Institute for Complex Systems, University of Namur, Namur, Belgium; 2 Department of Mathematics, Imperial College London, London, United Kingdom; 3 EPSRC Centre for Mathematics of Precision Healthcare, Imperial College London, London, United Kingdom; King’s College London, UNITED KINGDOM

## Abstract

Many collective phenomena in Nature emerge from the -partial- synchronisation of the units comprising a system. In the case of the brain, this self-organised process allows groups of neurons to fire in highly intricate partially synchronised patterns and eventually lead to high level cognitive outputs and control over the human body. However, when the synchronisation patterns are altered and hypersynchronisation occurs, undesirable effects can occur. This is particularly striking and well documented in the case of epileptic seizures and tremors in neurodegenerative diseases such as Parkinson’s disease. In this paper, we propose an innovative, minimally invasive, control method that can effectively desynchronise misfiring brain regions and thus mitigate and even eliminate the symptoms of the diseases. The control strategy, grounded in the Hamiltonian control theory, is applied to ensembles of neurons modelled via the Kuramoto or the Stuart-Landau models and allows for heterogeneous coupling among the interacting unities. The theory has been complemented with dedicated numerical simulations performed using the small-world Newman-Watts network and the random Erdős-Rényi network. Finally the method has been compared with the gold-standard Proportional-Differential Feedback control technique. Our method is shown to achieve equivalent levels of desynchronisation using lesser control strength and/or fewer controllers, being thus minimally invasive.

## Author summary

Synchronisation plays an important role in most of the neuronal activities and in particular in the control of the motor system. However, due to biochemical dysfunction in the brain activity, an abnormal and excessive synchronisation may occur being responsible for severe symptoms of several neurological diseases. For the case of Parkinson’s disease, for instance, an insufficient dopamine production in the basal ganglia causes rigidity or continuous tremors. In the case of epilepsy instead, imbalance between excitation and inhibition causes strong unpredictable seizures. Several neurostimulation techniques have been developed with the aim to control and relieve the symptoms as alternatives to oral medication. In this line of research, we propose a new method which has the property of being as little invasive as possible, in the number of electrodes needed and the strength of the current applied, while still controlling the symptoms. It is based on the consideration that neuronal patches resemble a set of phase-coupled oscillators which dynamics can be described by the celebrated Kuramoto model. The control technique we employ is inspired by a Hamiltonian formulation of the Kuramoto model. To verify the effectiveness of our method, we test it in a more realistic model of coupled neuronal patches described by the Stuart-Landau equations. Numerical simulations validate our approach.

## Introduction

Synchronisation is one of the key mechanisms responsible for self-organisation and emergence in living organisms [1–3]. Regular and periodic activity emerging from the collective behaviour of a set of interacting agents, has been noted to be crucial for the operation of many processes in living organisms [4, 5]. A prime example are the firing patterns of neuronal populations that form the basis of brain activity [6, 7] and their coordination among distributed mesoscopic neuronal populations [8] that ultimately controls our behaviour with an impressive and somehow mysterious accuracy [9]. It is therefore not surprising that defects or hypersynchronisation in neural firing patterns can lead to a host of neurological and psychiatric pathologies such as schizophrenia, Alzheimer’s disease, Parkinson’s disease and epilepsy [10–12].

One of the most conspicuous manifestation of neural hypersynchronisation are perturbation in the motor control systems. For example, a lack of dopamine in the basal ganglia is responsible for the uncontrolled and continuous tremors, rigidity and abnormal gait found in Parkinson’s disease (PD) [13]. Epilepsy is an even more striking example where strong and violent seizures occur unpredictably [14] and can be caused by an imbalance in neuronal excitation and inhibition [15]. While the exact causes of these diseases have yet to be elucidated [16], they share a common mechanism: a dysfunction of neuronal firing patterns. Being able to control and restore normal synchronisation patterns could alleviate or even eliminate the symptoms [17]. Long term drug treatments are the reality for most patients suffering from PD or epilepsy, with only partially satisfying results [13, 18–20] and the potential associated long term and side effects. An alternative to chemical treatments is neurostimulation, which induces a modulation of the neuronal activity in order to desynchronise the phase dynamics of neurons [21–24]. Our methods is to be applied in the framework of standard neurostimulation techniques [25–27] e.g. Deep Brain Stimulation (DBS) and is designed to render it as little invasive as possible, both by reducing the number of implanted electrodes and by using weaker applied currents. Although our methods may find clinical application in a number of diseases [28], we will focus on the control of focal epileptic seizure as an example. Our work is computational in spirit and aims at validating a control strategy using simple but effective computational models already in use in computational neuroscience. The usefullness of such approaches to investigate epilepsy to complement and guide experiments has recently been reviewed [29–31].

In this paper, we will focus on the theoretical description of a novel, minimally invasive, brain neurostimulation method. It is minimally invasive in the sense that with a similar number of electrodes as existing set ups, e.g. the Proportional-Differential feedback method [24] the strength signal needed to control the hypersynchronisation is set at the minimum enough level to desynchronise the neuronal patches (see Discussion at page 10 and Supplementary Material (SM)). This work lays the foundations of an applicable desynchronisation technique specifically aimed to control focal seizures, hypersynchronisation events in localised portion of the brain. We will first use the paradigmatic Kuramoto model [32, 33] (KM) to describe synchronisation in networks of small patches neurons that can be targeted by microelectrodes, and then extend our results to the more general Stuart-Landau model (SLM).

Neurons are commonly modelled using leak and fire model (LIF), and it has been shown that coherent synchronous behaviour can be obtained from very small patches of neurons [34]. The signal emerging from small neuronal patches can then be considered as phases of non-linearly coupled Kuramoto oscillators and their behaviour is indistinguishable from the more detailed LIF models. Networks of Kuramoto oscillators are particularly adapted to describe synchronisation pattern in neuronal patches: neuronal patches are connected with axonal tracts, forming a network of cells and they can synchronise easily their activation; i.e., neurons are able to synchronise even when operating in a weakly coupled regime. Indeed the parameter responsible for the interaction among neurons can, without any loss of generality, be considered small.

The method presented here simplifies the theoretical control term introduced in [35] to make it operational and show the potential for implementation. With this method, we can reduce the system wide phase synchronisation, or phase-locking, of nonlinearly coupled Kuramoto oscillators. The core mechanism brings the coupling between neurons patches below a certain critical value where partial synchronisation remains, but the system does not hypersynchronise. The magnitude of the control term, even when activated, is much smaller than the interaction among the patches and so minimally affects their activity. Once the coupling among the neurons is strong enough and the system is hypersynchronised, the control term naturally kicks in and induces a desynchronisation of the neuronal dynamics with the consequent suppression of the hypersynchronised behaviour. Keeping the control parameter at its lowest possible value both in the phase-unlocked and in phase-locked regime is important to avoid any side effects such as hallucinations or hypersexuality, commonly observed in other neurostimulation methods due to the stimuli being too strong [36]. For this reason, the proposed procedure for controlling the onset of the symptoms, as in the case of epilepsy, is optimised to get the right balance between managing the seizures and being as little invasive as possible.

The basis of the control framework proposed in this work [35] is grounded in the well established Hamiltonian control theory [37–40], which relies on the Hamiltonian formulation of the synchronisation process proposed in [41]. However, this theoretical control procedure [41] assumes a complete knowledge of the observables of the system: the network topology, phase variable and, more importantly, all the interacting nodes must be directly controlled. This is clearly not directly applicable to the brain where in the best case we can only measure the local dynamics and control only with a very limited number of patches of neurons compared with the whole number of neurons involved. To tackle this problem, we hereby adapt the theoretical control in order to limit the number of necessary microelectrodes to achieve the desired level of control and at the same time reduce the amount of information required on the signal measured from the electrodes. In the following section, we will introduce the mathematical formalism which describes the synchronisation phenomenon and give a short presentation of the Hamiltonian control theory, we invite the interested reader to consult [35] for a detailed discussion. Then, we illustrate our method with neuronal desynchronisation in the framework of the Kuramoto model. Let us observe that the method developed in [35] has been proposed in the framework of unweighted networks, where all the oscillators interact with the same strength, given by the Kuramoto parameter *K*. However, our method can be straightforwardly extended to the wider class of weighted networks (see SM). We finish by extending our results to the more complex and realistic Stuart-Landau model, which has been used to reproduce brain activity in different settings describing various diseases [42]. Controlling abnormal synchronisation patterns in this model makes a strong point for the applicability of our method in real situations. We then conclude by summing up our results.

## Methods

### Neuronal patches modelled as nonlinear oscillators

Abnormal synchronisation of the neural activity is responsible for the symptoms of many neurological diseases. Despite the very different nature of various systems exhibiting synchronisation, the main features are quite universal and can thus be described using the paradigmatic Kuramoto model [32, 33, 43–45] of nonlinearly coupled oscillators. Interestingly, as we show now, the KM is the limit of the more general Stuart-Landau model [24, 46]. This model is well adapted to describe the normal form of a supercritical Andropov-Hopf bifurcation, which describes the switch from a stationary state to a periodic one—limit cycle—(and vice versa) according to a single bifurcation parameter:

$${\dot{z}}_{k}=(1+\iota \omega_{k}-|z_{k}{|}^{2})z_{k}+Z_{k},\;\text{where}\;Z_{k}=\frac{K}{N}\sum_{j=1}^{N}A_{kj}z_{j}\;.$$ (1)

Here the complex variable $z_{k}=\rho_{k}e^{\iota \phi_{k}}$ encodes the information about the amplitude *ρ*_*k*_ and the phase *ϕ*_*k*_ of the coupled oscillators and $\iota =\sqrt{-1}$ is the imaginary unity. The others terms are: the natural frequencies of the oscillators *ω*_*k*_, and are drawn from a symmetric, unimodal distribution *g*(*ω*), the coupling strength *K* and the symmetric adjacency matrix *A*_*kj*_ encoding the connections among the *N* oscillator (*A*_*kj*_ = *A*_*jk*_ = 1 if oscillators *k* and *j* are coupled and zero otherwise). Considering the real part of Eq (1) and assuming the amplitudes to be almost equal, i.e. *ρ*_*k*_ ∼ *ρ*_*j*_ for all *k* and *j* (this statement is true in a weakly coupled regime as the case of neuronal patches ensemble), we obtain that the angles *ϕ*_*k*_ evolve according to the Kuramoto model

$${\dot{\phi }}_{k}=\omega_{k}+\frac{K}{N}\sum_{j=1}^{N}A_{kj}\text{sin}\left(\phi_{j}-\phi_{k}\right)\;.$$ (2)

We remind the reader that the original Kuramoto model corresponds to an all-to-all coupling [32], *A*_*kj*_ = *A*_*jk*_ = 1, for all *k* ≠ *j*, *A*_*kk*_ = 0. The model can be rewritten using the order parameter [33]

$$Re^{\iota \Psi }=\frac{1}{N}\sum_{j=1}^{N}e^{\iota \phi_{j}}\;,$$ (3)

a macroscopic quantity that measures the strength of the synchronisation; if *R* ∼ 0, the oscillators are almost independent each other while if *R* ∼ 1 they are close to be phase-locked. Substituting the above definition in the original model we get the mean-field equation

$${\dot{\phi }}_{k}=\omega_{k}+KR\text{sin}\left(\Psi -\phi_{k}\right)\;.$$ (4)

Thus the oscillators are no longer directly coupled to each other, but to the mean field oscillator with phase Ψ.

### Hamiltonian control and the synchronisation problem

The KM is a dissipative system, however an *N* dimensional Hamiltonian system *H*(***ϕ***, **I**) written in angles variables ***ϕ*** = (*ϕ*_1_, … *ϕ*_*N*_) and actions variables **I** = (*I*_1_, …, *I*_*N*_), has been proposed recently [41] and embeds as particular orbits the ones of the KM

$$\begin{array}{ccc} H(\phi ,I) & = & \sum_{i}\omega_{i}I_{i}-\frac{K}{N}\sum_{i,j}A_{ij}\sqrt{I_{i}I_{j}}\left(I_{j}-I_{i}\right)\;\text{sin}\left(\phi_{j}-\phi_{i}\right)\\ & \equiv & H_{0}\left(I\right)+V\left(\phi ,I\right)\;, \end{array}$$

where *H*_0_ and *V* are defined by the rightmost equality. The previous model represents a class of systems able to describe the Lipkin-Meshkov-Glick (LMG) model in the thermodynamic limit [47] and of the Bose-Einstein condensate in a tilted optical lattice [48]. The temporal evolution of the angle-action variables is obtained from the Hamilton equations:

$${\dot{I}}_{i}=-\frac{\partial H}{\partial \phi_{i}}=-2\frac{K}{N}\sum_{j=1}^{N}A_{ij}\sqrt{I_{i}I_{j}}(I_{j}-I_{i})\;\text{cos}\left(\phi_{j}-\phi_{i}\right)$$ (5)

$${\dot{\phi }}_{i}=\frac{\partial H}{\partial I_{i}}=\omega_{i}+\frac{K}{N}\sum_{j=1}^{N}A_{ij}[2\sqrt{I_{i}I_{j}}\;\text{sin}\left(\phi_{j}-\phi_{i}\right)-\sqrt{I_{j}/I_{i}}(I_{j}-I_{i})\text{sin}\left(\phi_{j}-\phi_{i}\right)]$$ (6)

for *i* = 1, …, *N*. More precisely, one can define the invariant Kuramoto torus $T:=\{\left(I,\phi \right)\in R_{+}^{N}\times T^{N}:I_{i}=1/2\;\forall i\}$ and prove, to have a more detailed description of the model and of its properties. that the restriction of time evolution of the angles variables (*ϕ*_1_, … *ϕ*_*N*_) to this torus coincides with Eq (2). We refer the interested reader to [35, 41] for further details.

In [41], the authors have analytically proved and confirmed numerically that when the Kuramoto oscillators enter in a synchronised state, the dynamics of the actions close to the Kuramoto torus become unstable and exhibit a chaotic behaviour. Based on this result, our aim is to reduce the synchronisation in the KM (2) by controlling the Hamiltonian system *H*(***ϕ***, **I**) by adding a small control term able to increase the stability of the invariant torus T. Based on the previous remark this implies a reduction of the chaotic behaviour close to said torus, and thus impedes the phase-locking of the coupled oscillators. Let us rewrite the Hamiltonian in the form *H* = *H*_0_ + *V*, where *H*_0_ is the integrable part, i.e. the uncoupled harmonic oscillators, and *V* the non-linear term, namely the *KR*sin(Ψ − *ϕ*_*k*_) function in the KM, that can be considered as a perturbation of *H*_0_ because of the small magnitude of the parameter *K*. The main idea of Vittot and coworkers [37, 39] is to add to *H* a small control term $f∼O(K^{2})$, whose explicit form depends on *V*, in order to reduce the impact of the perturbation *V*, i.e. to increase the stability of the invariant torus. The size of *f* implies that the controlling procedure is much less invasive than other techniques generally used in control theory and is also able to give a rapid response to possible abnormal dynamics and more importantly, without any need for further measurement of the state of the system. Assuming a technical condition on the natural frequencies, namely ***ω*** = (*ω*_1_, …, *ω*_*N*_) to be not resonant, i.e. for all k∈Z\{0} then **k** ⋅ ***ω*** ≠ 0, one can straightforwardly compute the required control term *f*(***ϕ***, **I**). Let us observe that the theory by Vittot can also handle more general cases where such additional assumption is relaxed.

The embedding of the KM in the Hamiltonian system is based on the existence of the invariant torus T, which is no longer invariant for the controlled Hamiltonian *H*_0_ + *V* + *f*. Nevertheless, it is possible to provide an effective control by truncating the latter to its first term, such that the resulting controlled Hamiltonian system preserves the Kuramoto torus. One can thus transpose this information into the KM and achieve a control strategy:

$${\dot{\phi }}_{k}=\omega_{k}+KR\text{sin}\left(\Psi -\phi_{k}\right)+h_{k}\left(\phi_{1},\ldots ,\phi_{N}\right)\;,$$ (7)

where *h*_*k*_(*ϕ*_1_, …, *ϕ*_*N*_) is the contribution of the control *f* to the angles dynamics and is explicitly given by:

$$\begin{array}{c} h_{k}\left(\phi_{1},\ldots ,\phi_{N}\right)=-\frac{K^{2}}{4N^{2}}[\sum_{j}A_{kj}\;\text{cos}\left(\phi_{j}-\phi_{k}\right)\sum_{l}\frac{A_{kl}}{\omega_{l}-\omega_{k}}\text{cos}\left(\phi_{l}-\phi_{k}\right)+\\ \;+\sum_{j}\frac{A_{kj}}{\omega_{j}-\omega_{k}}\text{sin}\left(\phi_{j}-\phi_{k}\right)\sum_{l}A_{kl}\text{sin}\left(\phi_{l}-\phi_{k}\right)+\\ \;-\sum_{l}(A_{kl}\;\text{cos}\left(\phi_{k}-\phi_{l}\right)\sum_{j}\frac{A_{jl}}{\omega_{j}-\omega_{l}}\text{cos}\left(\phi_{j}-\phi_{l}\right)+\frac{A_{kl}}{\omega_{k}-\omega_{l}}\text{sin}\left(\phi_{k}-\phi_{l}\right)\sum_{j}A_{jl}\text{sin}\left(\phi_{j}-\phi_{l}\right))]\;, \end{array}$$ (8)

where, with a slight abuse of notation, we used the same letter to denote the new controlled angular variable. The truncation to the first order of the control term *f* is justified by the Hamiltonian perturbation theory. Moreover the smaller the perturbation parameter *K*, being $f∼O(K^{2})$, the better the approximation. The details of the derivation of formula (8) can be found in [35].

To simplify the previous equation, let us introduce a second modified local order parameter that depends on the node index:

$${\tilde{R}}_{k}e^{\iota \Psi_{k}}=\frac{1}{N}\sum_{j=1}^{N}\frac{e^{\iota \phi_{j}}}{\omega_{j}-\omega_{k}}\;.$$ (9)

Under the hypothesis of an all-to-all coupling and a straightforward computation, the control term can be rewritten as:

$$h_{k}\left(\phi_{1},\ldots ,\phi_{N}\right)=-\frac{K^{2}}{4}[R{\tilde{R}}_{k}\text{cos}\left(\Psi -\Psi_{k}\right)-B_{k}]\;,$$ (10)

where $B_{k}$ is defined by

$$B_{k}=\frac{1}{N}\sum_{l}\text{cos}\left(\phi_{k}-\phi_{l}\right)\text{cos}\left(\Psi_{l}-\phi_{l}\right){\tilde{R}}_{l}+\sum_{l}\frac{\text{sin}(\phi_{k}-\phi_{l})}{\omega_{k}-\omega_{l}}\text{sin}\left(\Psi -\phi_{l}\right)R\;.$$

## Results

### Effective desynchronisation of the phases of coupled neurons

Before we enter into the technical details of the proposed method, let us first comment on the analytic result obtained above and discuss its advantage with particular attention to the control of the onset of abnormal synchronisation. As already anticipated earlier in this paper, our principal aim is to develop a novel method to lower the synchronisation level of the neuronal patches situated in regions responsible for causing symptomatic behaviour. However, current neurostimulation techniques achieving this goal are often strongly invasive in terms of its strength. Our aim is to optimise the control strategy by letting the control to act only when necessary and with minimal magnitude. This means that, although the control is always present, it should dynamically “switch on”, i.e. achieve a strength comparable to the one of the signal, when the seizures start and again dynamically “switch off”, i.e. become negligible with respect to the signal) during the normal neuronal regime. This is exactly what the proposed control term (8) does; the two main contributions to the control are the prefactor *K*^2^ and the denominators containing the differences of natural frequencies *ω*_*j*_ − *ω*_*k*_, which are of the order of the width of the frequency distribution *g*(*ω*). Because the critical value of the coupling strength *K*_*c*_ (< 1), the value for which the system exhibits a synchronised state, is of the order ∼ *g*(*ω*) [33], the control term becomes of order *K* in the critical regime, exactly when it is necessary to reduce the synchronisation. On the other hand, during the normal regime the control size is much smaller than the critical one, *K*^2^ ≪ *K*_*c*_, and in consequence the method can be considered to be minimally invasive.

Let us now come back to the theoretical control term (10) and prove that one can realise it as an operational control strategy. The first observation is that the latter requires the control of all neuronal patches, and this is impossible to be achieved in a realistic situation. The second observation is that the control demands the exact knowledge of the connectivity of all the interacting cells. From a practical point of view we *a priori* know that in order to control the synchronisation we must interfere with the neuronal dynamics, by sending an electrical signal through a microelectrode inserted into a suitable zone of the brain. For this reason the main dilemma inherent with all neurostimulation methods is how to be as little invasive as possible but at the same time as efficient as possible? To give a possible solution to this issue we will simplify the formula (10) to fit our goal of having an operational control. We will show that we can obtain a desynchronisation effect using a limited number of controlling microelectrodes, as good as the one involving the control of all the patches. We will work under the hypothesis that the interaction network allows easy global interactions while having a strong local connectivity, i.e. a small-world type of architecture [49]. This assumption is justified by experimental observations [50] which describe mesoscopic brain networks as *small-world*. Although the control method proposed here operates at a much smaller scale than the ones considered in [50], there are compelling evidence [51] and models [52] that a form of self-similarity of brain circuitry and function is present, and thus what is observed at a macroscale can be inferred to be similar at a smaller scale. For this reason we believe that the theory previously developed under the assumption of all-to-all coupling, can be applied to a more general network topology, without substantially modifying the resulting dynamics. In this respect, the microelectrodes are supposed to be positioned, for instance as best as possible in the epileptic foci and each of them to directly control a zone which includes a certain number of neurons, optimising the efficiency of the control.

The first observation is that the second term in Eq (10), $B_{k}$, is often much smaller than the first one; mathematically this can be understood because this term involves averages of products of oscillatory functions that can thus compensate each other. The second observation is that one can hardly compute the local phase Ψ_*k*_ using a limited number of microelectrodes sampling few neurons, we then decide to replace the latter with the neuron phase *ϕ*_*k*_. The last point concerns the term ${\tilde{R}}_{k}$ whose computation requires the knowledge of the phases and the natural frequencies of all the neurons. In a real implementation of the control strategy this requirement is too stringent to be achieved, we thus decided to replace it with a term, ${\hat{R}}_{k}$, computed using information obtained only from the neuronal patches where the microelectrodes are implanted in

$$\forall k=1,\ldots ,M\;{\hat{R}}_{k}e^{\iota {\hat{\Psi }}_{k}}=\frac{1}{M}\sum_{j=1}^{M}\frac{e^{\iota \phi_{j}}}{\omega_{j}-\omega_{k}}\;.$$ (11)

In the previous formula, we assumed the ordering the neuronal patches *j* to be such that the first *M* are the ones upon which the microelectrodes are implanted. We are aware of the impact of these working assumptions, nevertheless the justification of these choices is obtained *a posteriori* by observing that the effective control performs very well. In conclusion the proposed local control strategy is given by:

$$\forall k=1,\ldots ,M\;{\hat{h}}_{k}\left(\phi_{1},\ldots ,\phi_{M}\right)=-\frac{\gamma }{4}K^{2}R{\hat{R}}_{k}\text{cos}\left(\Psi -\phi_{k}\right)\;,$$ (12)

where we stressed again the dependence of such control term only onto the *M* neuronal patches upon which the microelectrodes are set in. Let us observe that we added a free parameter *γ* to take into account the direct action on a small number of nodes, *M* ≪ *N*, and the imperfectly known network structure. In particular *γ* can be set equal to the ratio of the average connectivity with the maximum possible number of links, which is a macroscopic parameter that can be known with good precision in advance. In conclusion let us observe that the local control term is built using a cosine function which is nothing but the coupling term in the KM (2) delayed by a quarter of its period *T*. We thus recover the empirical rule proposed by [22] consisting in the re-injection in the microelectrodes used in the DBS of the measured signal delayed by one fourth of its period. The operational control of a given neuronal patches is the following: compute the signal from a given neuron through a microelectrode, delay the signal by *T*/4, multiply it by $\gamma K{\hat{R}}_{k}/4$, where ${\hat{R}}_{k}$ is computed using a limited number of signals from neurons where the microelectrodes are inserted, and re-inject the new signal in the initial neuron using the same microelectrode. In this way the latter will desynchronise and break away from the whole system acting as a single giant oscillator.

We however observe that this is not enough to desynchronise the whole system, but only the controlled nodes where the microelectrode is placed. Because we want to limit the number of implanted electrodes, this strategy will not be able to sufficiently reduce the symptoms. To achieve our goal, it is thus necessary to indirectly influence the behaviour of the other neurons. This can be done be noticing that a microelectrode controlling a given node produces an electromagnetic field potential [53, 54]. To be more specific, let us denote with $S_{k}^{stim}$ the stimulation signal generated on the position of the *k*-th neuron by the potential produced by the microelectrodes located in all the controlled neuronal patches, mathematically:

$$S_{k}^{stim}\left(\phi_{1},\ldots ,\phi_{M}\right)=c_{s}\sum_{l=1}^{M}e^{-2r_{kl}}{\hat{h}}_{l}\left(\phi_{1},\ldots ,\phi_{M}\right)\;;$$ (13)

where *r*_*kl*_ and *c*_*s*_ are respectively the distance of node *k* from the origin of the electromagnetic field *l*, and the strength of the potential which in our case is taken to be *c*_*s*_ = 1, finally *M* ≪ *N* is the number of directly controlled nodes.

**Fig 1:**
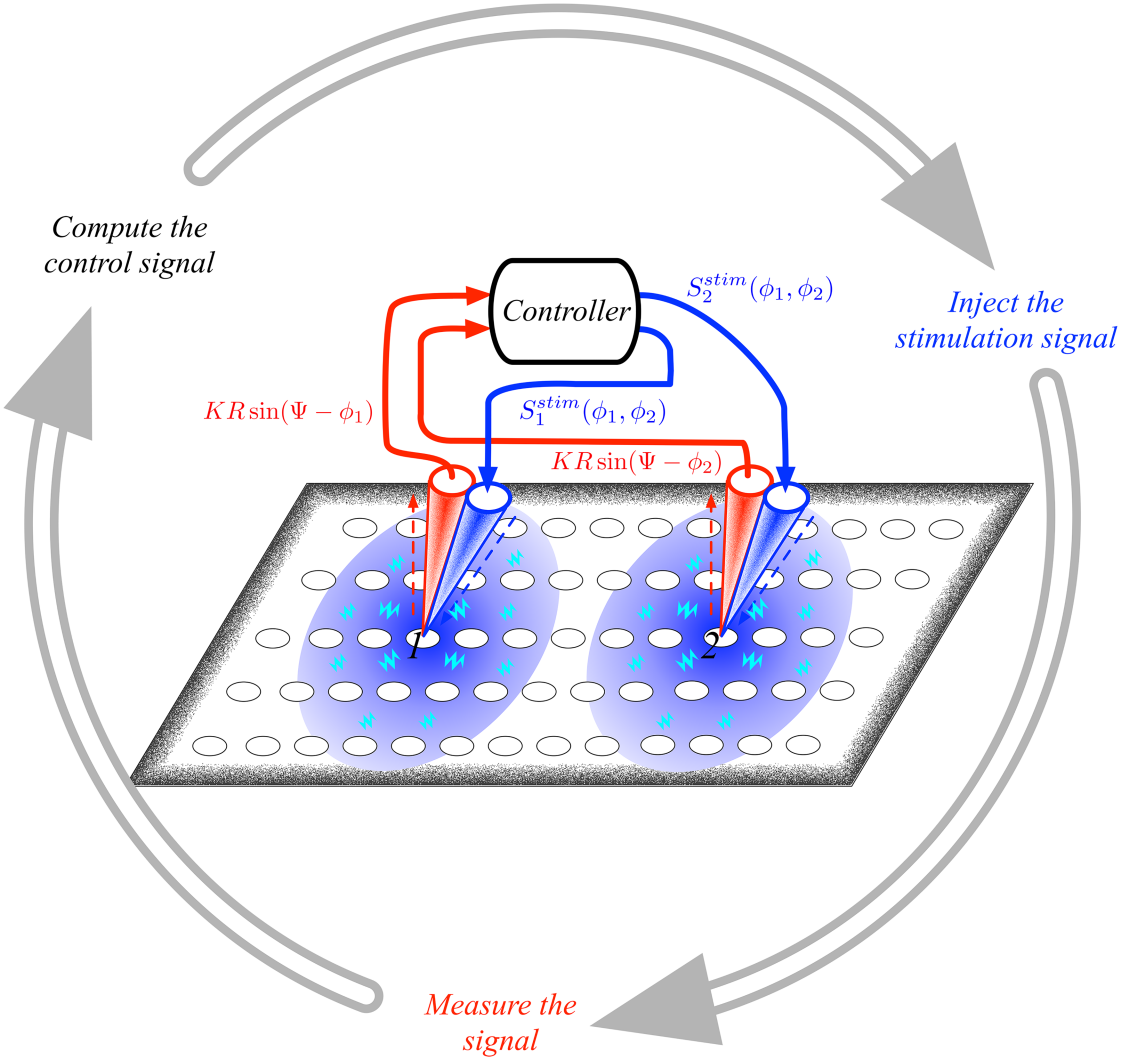
A schematic description of the control strategy. An array of neuronal patches (white circles) is controlled using *M* = 2 microelectrodes (red-blue cones). The signals, *KR*sin(Ψ − *ϕ*_*i*_), *i* = 1, 2, acquired by each microelectrode are passed (red arrows) to the controller that computes the control signals, $S_{i}^{stim}\left(\phi_{1},\phi_{2}\right)$, *i* = 1, 2, which are in turn (blue arrows) injected back to the neuronal patches 1 and 2. This determines an electromagnetic field which possesses a spatial extension whose strength decays with the distance from the injection point (large circles with shaded of blue). In this way nearby neuronal patches are also influenced but in a weaker fashion. The mechanism is presented as a sequence of steps repeated cyclically (external grey circular arrows), however under the assumption that the measurement and the computation of the control and the injection are very fast with respect to the natural time scale of the underlying system, this process can be considered to be instantaneous and thus acting directly on the evolution of the system without any delay (see Eq (14)).

In conclusion the proposed control strategy will modify the activity of all the *N* neurons as follows:

$${\dot{\phi }}_{k}=\omega_{k}+KR\text{sin}\left(\Psi -\phi_{k}\right)+S_{k}^{stim}\left(\phi_{1},\ldots ,\phi_{M}\right)\;,$$ (14)

let us observe that if the *k*–th neuronal patch has a microelectrode implanted into it, the rightmost term can be rewritten as $c_{s}{\hat{h}}_{k}\left(\phi_{1},\ldots ,\phi_{M}\right)+c_{s}\sum_{l\neq k}^{M}e^{-2r_{kl}}{\hat{h}}_{l}\left(\phi_{1},\ldots ,\phi_{M}\right)$, namely the direct control term plus the electromagnetic field generated by the remaining *M* − 1 microelectrodes, while if the *k*–th neuronal patch doesn’t have any microelectrodes it will feel the resulting electromagnetic field. A schematic illustration of the proposed control method is given in Fig 1.

**Fig 2:**
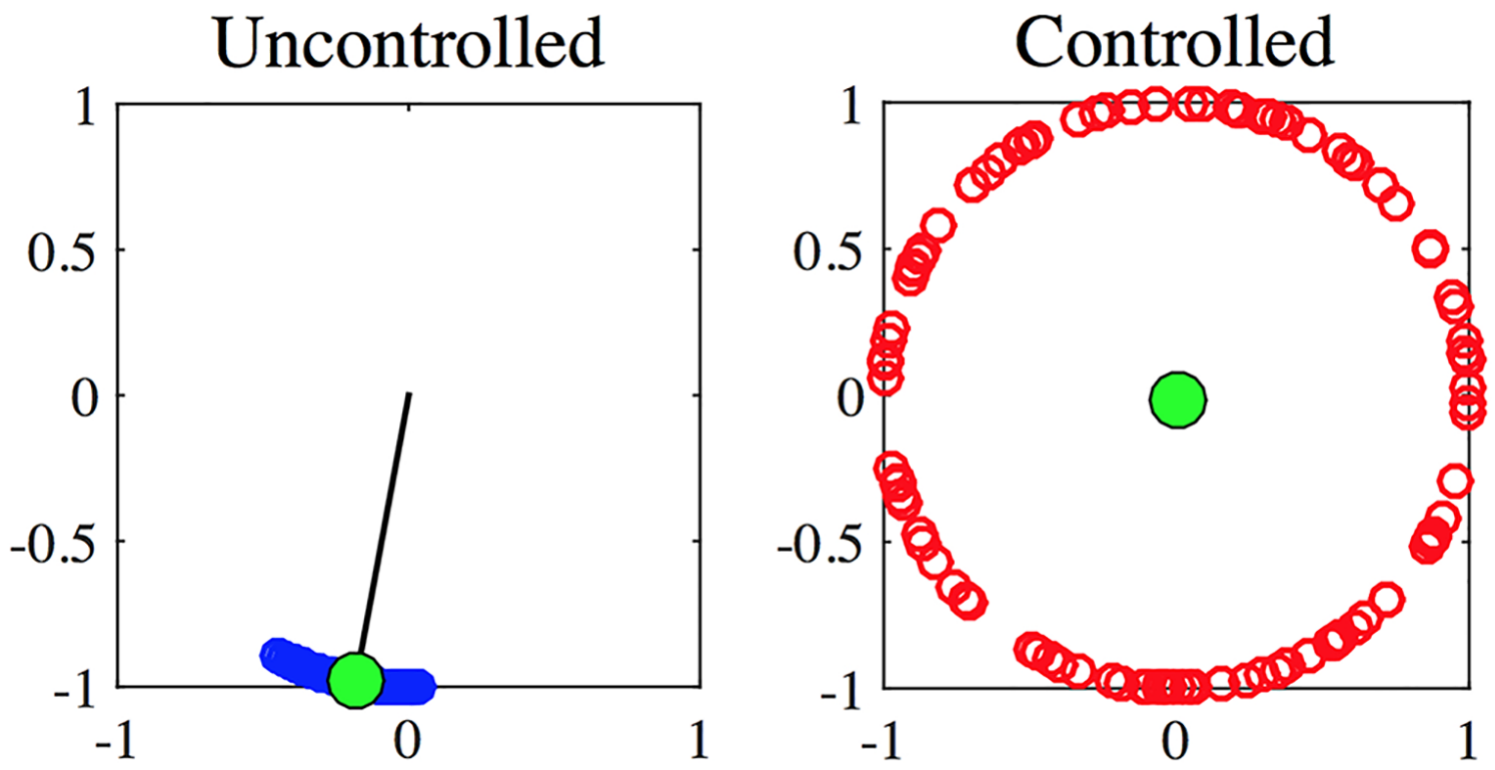
A snapshot of the Kuramoto dynamics at a generic time. *N* = 100 oscillators (circles) are drawn on the unitary circle, their angular position is given by the oscillator phase. The dynamical behaviour presented in panel a) corresponds to the uncontrolled phase-locked regime for a coupling parameter *K* = 0.5. In panel b) we report, for the same coupling parameter, the controlled case obtained acting on *M* = 20 oscillators and *γ*/4 = 4.25, resulting in a desynchronised behaviour. The underlying network is a Newman-Strogatz small-world network [55] with parameter *p* = 0.85. The green circle represents the Kuramoto order parameter, its angular position is given by the angle Ψ while its distance from the origin is *R*.

In Fig 2, we report the results of a generic simulation for the Kuramoto model; an oscillator is represented by a circle laying on the unit circle whose angular coordinate is given by the oscillator phase. The green circle identifies the Kuramoto order parameter, its angular position being given by Ψ while the distance from the origin, the black segment (clearly visible on the panel a)), represents *R*. Let us observe that the longer such segment is, i.e. the larger *R*, the stronger the synchronisation of the oscillators is. This can be clearly appreciated on panel a) where most of the circles are very close to the green one. On the other hand (see panel b)) one can observe that in the case of non-synchronisation the oscillators are quite uniformly distributed on the circle, resulting in *R* ∼ 0. The network chosen for coupling the 100 oscillators is a Newman-Watts small-world [55].

The Newman-Watts network is a well known and widely used generating model for complex networks, and exhibits the small-world property for a determined set of parameters; it differs from the other widely used model of small-world network, i.e. the Watts-Strogatz [49], mainly because the resulting network is always connected, and thus does not have isolated nodes. This is extremely important in our case where the neuronal patches by definition form a connected structure. The model contains a single parameter, *p* ∈ [0, 1], which determines the density of the network; indeed the network generation starts from a 1D regular lattice with coordination number 2*k*, i.e. each node is connected to its first *k* neighbours counted clockwise and *k* counterclockwise, then each couple of unconnected nodes is considered and with probability *p* a link is added. In the limit *p* → 1 many links can be potentially added and the network can become very dense; in the opposite case the network is sparse and very similar to the 1D regular lattice backbone.

In Fig 2 we can clearly observe that in the uncontrolled KM the oscillators tend to synchronise for the chosen coupling parameter. They almost all have the same phase (see panel a)), as *K* = 0.5 is larger than the critical parameter, here *K*_*c*_ ≈ 0.4. On the other hand, for the same value of the coupling parameter, but applying the effective control using *M* = 20 oscillators and *γ*/4 = 4.25, the behaviour is completely different, the oscillators are almost uniformly distributed on the unit circle (see panel b)), corresponding to a desynchronised system.

Let us emphasise that the results reported in Fig 2 are another *a posteriori* proof of the goodness of the control given by Eq (14). Indeed, despite the latter, as well as the theory presented in [35], has been developed under the assumption of all-to-all coupling, it works perfectly on a different underlying topology such as the Newman-Watts; in the SM we present a complete analysis of the role of the parameter *p* in the desynchronisation problem. Moreover in the SM we have improved the control strategy and extended it to handle weighted complex networks, and so make a further step towards empirical topologies.

The control strategy we proposed depends on two main parameters: the number of controlled microelectrodes *M* and the strength of the injected signal *γ*. Intuitively large values are associated to an efficient control for both parameters and thus to a reduction/suppression of the abnormal synchronisation, but with the drawback of being invasive; many microelectrodes have to be implanted and the strength of the signal could induce undesired collateral effects. Let us observe that under the assumption of all-to-all coupling and homogeneous interaction strengths among the oscillators, the results above are indistinguishable and thus the spatial layout of the microelectrodes does not matter. The same result seems to hold in the case of more complex coupling (see SM), for these reasons, we decided to position the microelectrodes uniformly at random among the oscillators.

**Fig 3:**
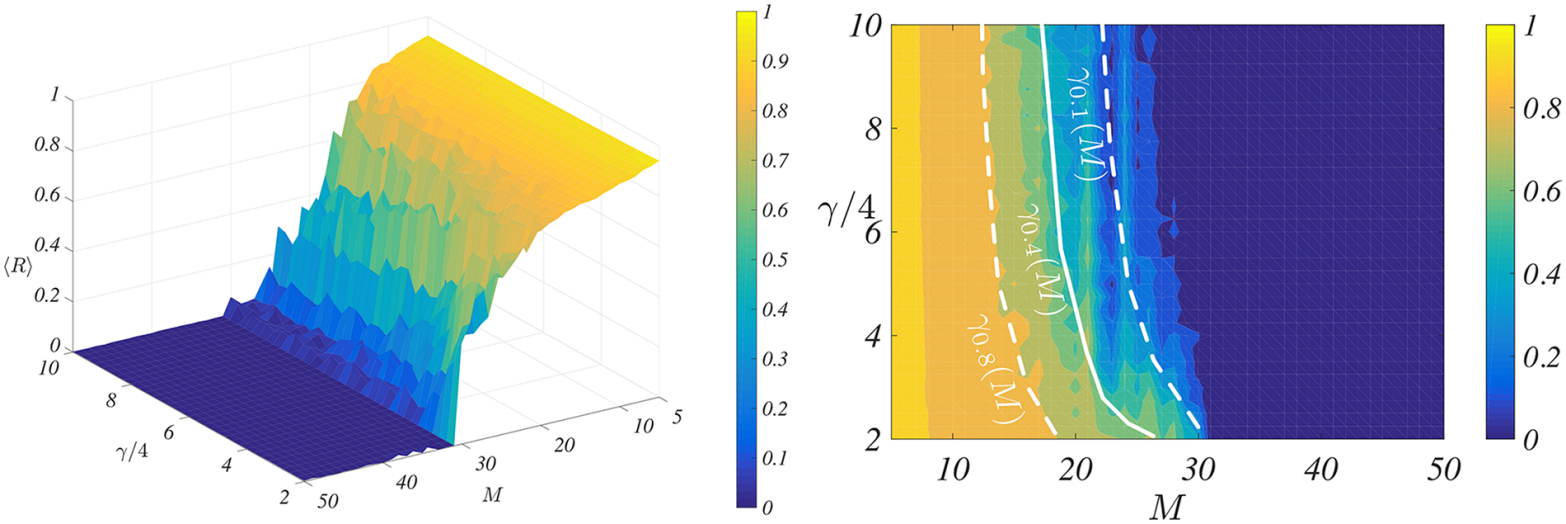
The Kuramoto order parameter as a function of number of microelectrodes and the strength of the control. Left panel: for each couple (*M*, *γ*/4) ∈ [5, 50] × [2, 10] we numerically simulate the controlled system (12) involving *N* = 100 oscillators interconnected using an all-to-all topology, with coupling parameter *K* = 0.5 and frequencies *ω*_*i*_ drawn from a normal distribution N(1,0.1). Each point is the average over 50 independent realisations (different initial conditions but same frequencies). Right panel: a different visualisation of 〈*R*〉 allowing to emphasise the relation *γ*_*r*_(*M*), *r* = 0.8 (dashed curve), *r* = 0.4 (dotted curve) and *r* = 0.1 (solid curve); let us stress that the latter curves have been draw with a visual guide scope and have not been analytically determined.

To understand the impact of *M* and *γ* and possibly determine an optimal range of values we performed a series of numerical simulations. In Fig 3 we report (left panel) the averaged (over 50 independent repetitions) Kuramoto order parameters, 〈*R*〉, for the controlled system (12) as a function of the parameters *M* and *γ*/4. One can observe that for large enough *M* ≳ 33, the control is able to completely suppress the synchronisation, 〈*R*〉 ∼ 0, for all values of *γ*/4. For intermediate values, 15 ≲ *M* ≲ 30, there exists a non trivial relation *γ*_*r*_(*M*) (see right panel) such that if *γ*/4 ≥ *γ*_*r*_(*M*) then the control can achieve a partial desynchronisation, 〈*R*〉 ≤ *r* (here *r* ∈ (0, 1) is a parameter defining the amount of partial desynchronisation present in the system). Finally for too small values of *M* ≲ 10, the proposed strategy is not able to reduce the synchronisation for any tested values of *γ*.

To support the claim that our method is minimally invasive, both in terms of the number of microelectrodes needed and the strength of the signal applied, we compared it with the Proportional-Differential Feedback technique (PDF) [24], whose capability to suppress hypersynchronisation has been already proved. In short, the main idea of the PDF is to split the population of *N* oscillators into two groups: a first group made by *N*_1_ elements whose signal is measured in time; and a second group, containing the remaining *N*_2_ = *N* − *N*_1_ oscillators, that will receive the feedback signal which is proportional to the mean-field signal of the *N*_1_ first oscillators, with a proportionality parameter *P* > 0, and to the derivative of the same signal, with a proportionality parameter *D* ≥ 0. We refer the interested reader to [24] and to the SM for a more detailed presentation of the PDF.

**Fig 4:**
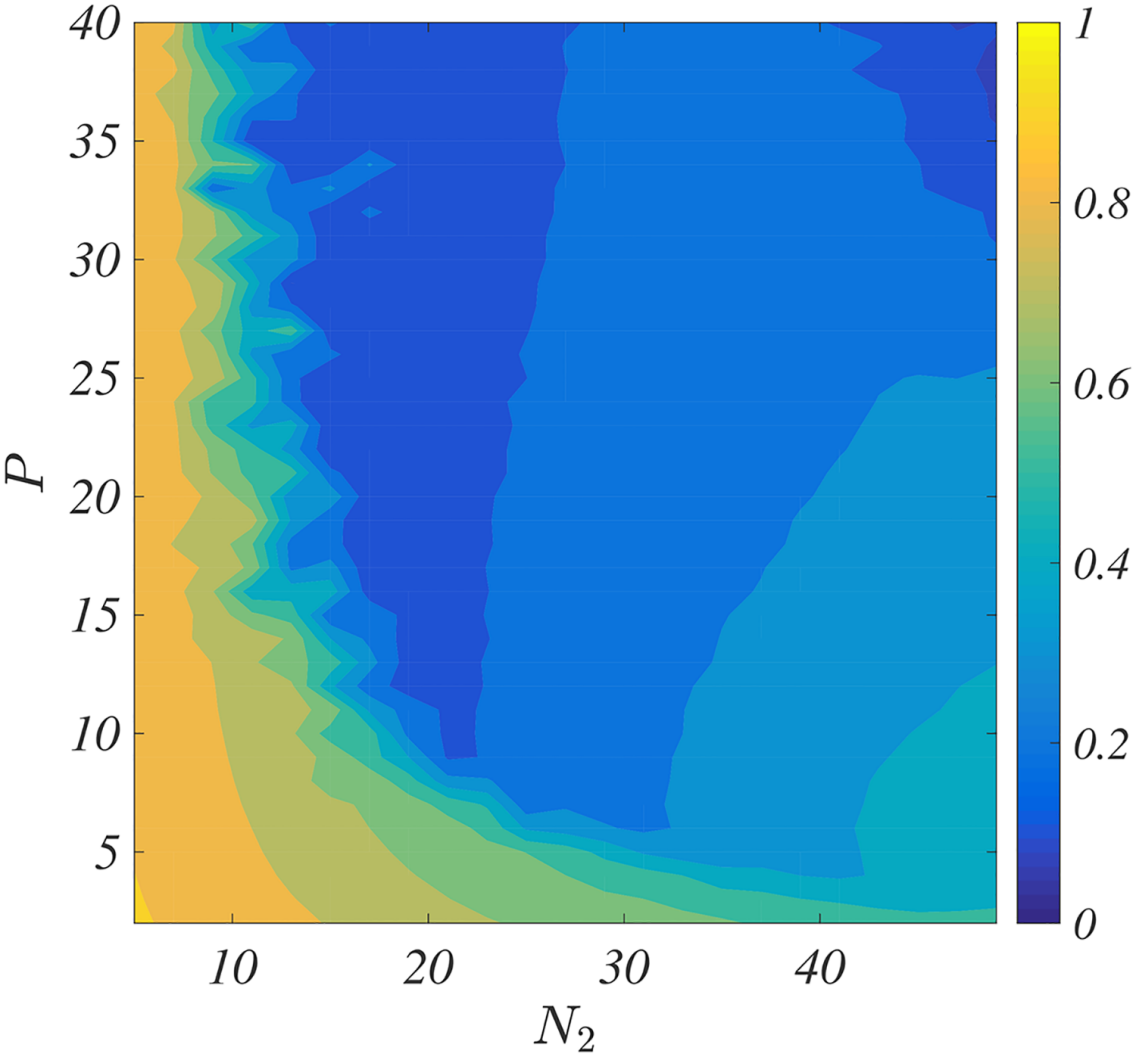
The role of *N*_2_, *P* and *D* on the desynchronisation for the PDF-control. The average Kuramoto order parameter 〈*R*(*t*)〈 is reported as a function of number of controlled oscillators, *N*_2_, and the control strength, *P*, for *D* = 10. For each couple (*N*_2_, *P*) ∈ [5, 50] × [2, 40], we numerically simulate the PDF-controlled system on the KM involving *N* = 100 oscillators coupled using an all-to-all scheme; each point being the average of 25 independent realisations. The coupling parameter is *K* = 0.5 and the natural frequencies are drawn from a normal distribution N(1,σ), *σ* = 0.01 and the initial angles uniformly randomly drawn in [0, 2*π*].

The PDF, like our method, is thus essentially based on two parameters, the number of controllers *N*_2_ and the strength of the feedback signal *P* + *D*, a comparison among the two methods is thus straightforward: *N*_2_ = *M* and *P* + *D* = *γ*/4. We have thus chosen as benchmark the KM composed by *N* = 100 oscillators coupled with an unweighted all-to-all network. We have numerically computed the asymptotic synchronisation state for several values of the parameters, measured with the Kuramoto order parameter $R=|\sum_{j}\;e^{\iota \phi_{j}}|/N$, and averaged the over several independent realisations. The results presented in Fig 4 (see also Fig. D in S1 Text) should be compared with the ones of Fig 3 (the same colour code has been used to help the comparison). At first glance both methods exhibit the same behaviour, for a small number of controllers one needs a large control strength, *P* + *D* or *γ*/4, to remove/reduce the synchronisation, and below a certain values of *N*_2_ or *M* desynchronisation cannot be achieved. However looking at the values of *P* + *D* versus *γ*/4 we realise that the former are 5 times larger. Indeed *P* + *D* ranges from 10 to 50 while *γ*/4 in the interval [2, 10]. This means that for the same number of controllers our method requires a much weaker signal strength or that for a fixed control strength we can achieve a desynchronisation level with a smaller number of implanted microelectrodes.

**Fig 5:**
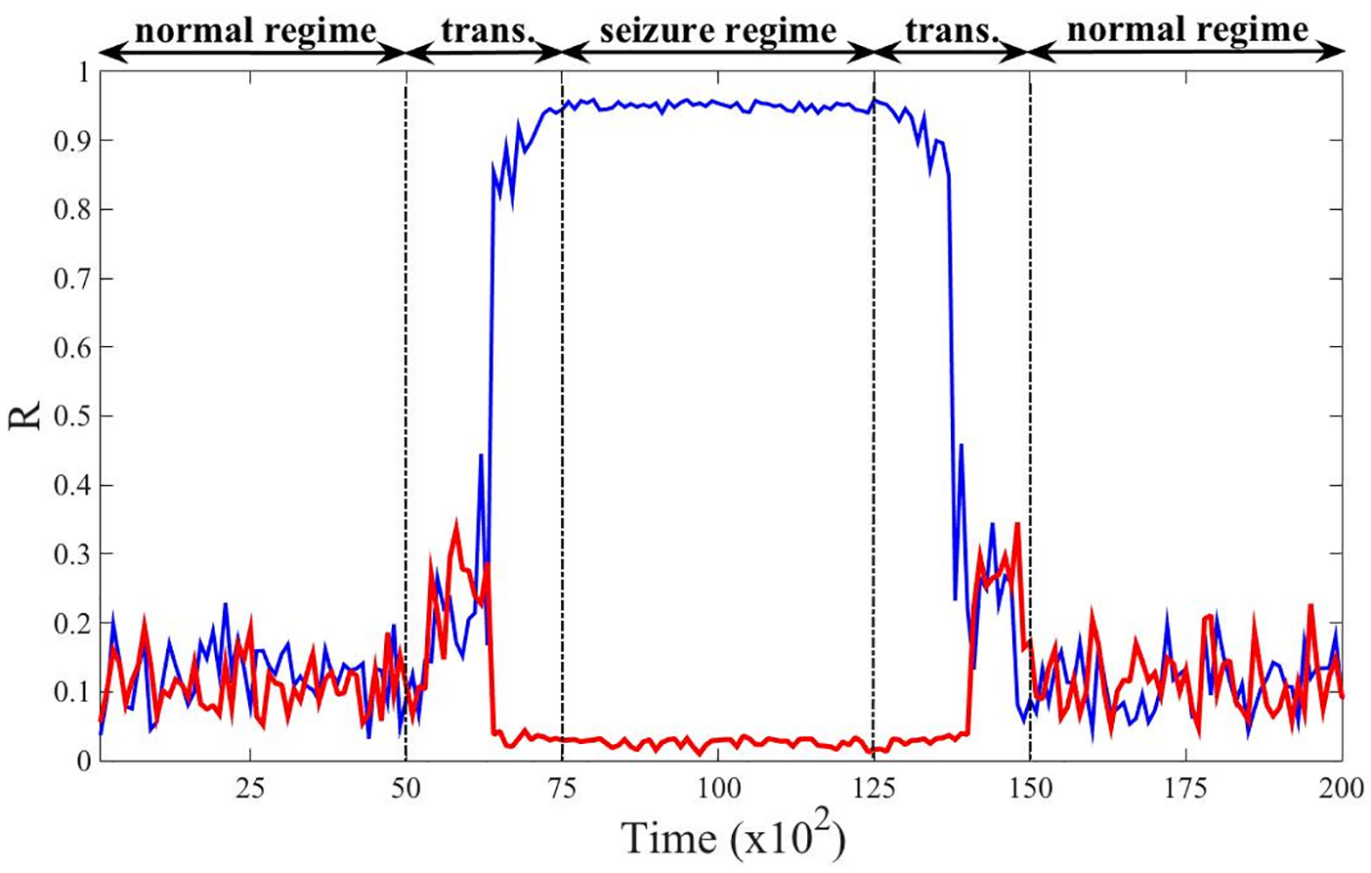
Onset of an epileptic seizure in the Kuramoto-like neuron population and the outcome of the controlled system. We represent the order parameter *R* (blue curve for the KM model and red curve for the controlled version) as a function of time for *N* = 100 coupled oscillators linked using a Newman-Watts small-world network [55] with parameter *p* = 0.85 and *M* = 20 microelectrodes for the controlled case (*γ*/4 = 4.25). To smooth the results, each curve is the average over 20 independent realisations. We assume *K* to be small in the interval [0, 5000] fluctuating around the average value 0.1, during this period of time both systems behave similarly and do not exhibit synchronisation. Then we assume the coupling parameter starts to increase, *t* ∈ [5000, 7500], to eventually remain quite large, 0.5 on average, for *t* ∈ [7500, 125000]; we can observe that the KM falls in a synchronised state while the controlled one still does not exhibit synchronisation. Once the coupling parameter decreases and fluctuates again around a small value, 0.1, both systems recover the same non-synchronised state.

To mimic the onset of an epileptic seizure in the brain and the action of the proposed control strategy, we realise the following numerical experiment: using *N* = 100 neurons connected using a Newman-Watts small-network, firstly without control (reference case) and then controlled using *M* = 20 microelectrodes and *γ*/4 = 4.25. In both cases, during a given period of time, [0, 5000], we numerically solve the KM with a small control parameter fluctuating in time to mimic the physiological fluctuations one can observe in neuron; more precisely every Δ*t* = 100 time units, we draw a value for *K* from a uniform distribution with support [0.05, 0.15], and thus with average 0.1, and we follow the model dynamics during Δ*t* time units. Let us observe that the coupling parameter is smaller than the critical one, *K*_*c*_ ≈ 0.4, and thus the system, for both the reference case and the controlled one remains in a non-synchronised state. This can be appreciated from Fig 5 where we plot the order parameter *R* as a function of time for the uncontrolled (blue) and controlled (red) KM. Observe moreover that both systems behave very similarly (the curves are very close), hence the control, even if present, is not changing the dynamics when not needed.

Then we assume that the coupling parameter quickly increases and then fluctuates around a large value, mimicking a seizure. Mathematically we assume that during the time interval [5000, 7500] every Δ*t* = 100 time units, we draw a value for *K* from a uniform distribution whose average grows linearly in time from 0.1 at *t* = 5000 to reach 0.5 for *t* = 7500, while in the interval [7500, 125000] the coupling parameter is drawn from a uniform distribution with support [0.55, 0.65], and thus with average 0.6. The results of the numerical simulations are striking, after a short transient time the uncontrolled system (blue curve) almost fully synchronises, *R* is very close to 1, while the controlled one remains in the non-synchronised phase, with *R* very close to 0. Of course when the coupling parameter starts to decrease to eventually reach again a small average value, the original reference system and the controlled one both exhibit again a non-synchronised behaviour.

### Effective desynchronisation of coupled neuronal patches

In the previous section we built an operational control scheme able to effectively reduce the level of phase-locking in the phases of the neurons described by the Kuramoto model, even for large values of the coupling parameter. As previously stated, the KM can be derived from the more general Stuart-Landau model, it is then natural to try to extend the control strategy to act directly on the Stuart-Landau system, self-consistently defined in terms of the control used for the Kuramoto model.

Let us consider Eq (1) under the assumption of all-to-all connection with the additional term $Z_{k}^{ctrl}=-\iota \frac{K}{4}{\tilde{R}}_{k}Z$, where $Z=\frac{K}{N}\sum_{j=1}^{N}z_{j}$, namely

$${\dot{z}}_{k}=(1+\iota \omega_{k}-|z_{k}{|}^{2})z_{k}+Z+Z_{k}^{ctrl}\;.$$ (15)

Assuming the amplitudes to be very close each other, one can easily prove that the phase of the complex variable $z_{k}=\rho_{k}e^{\iota \phi_{k}}$ in Eq (15) evolves according to the controlled Kuramoto system, see Eq (7).

**Fig 6:**
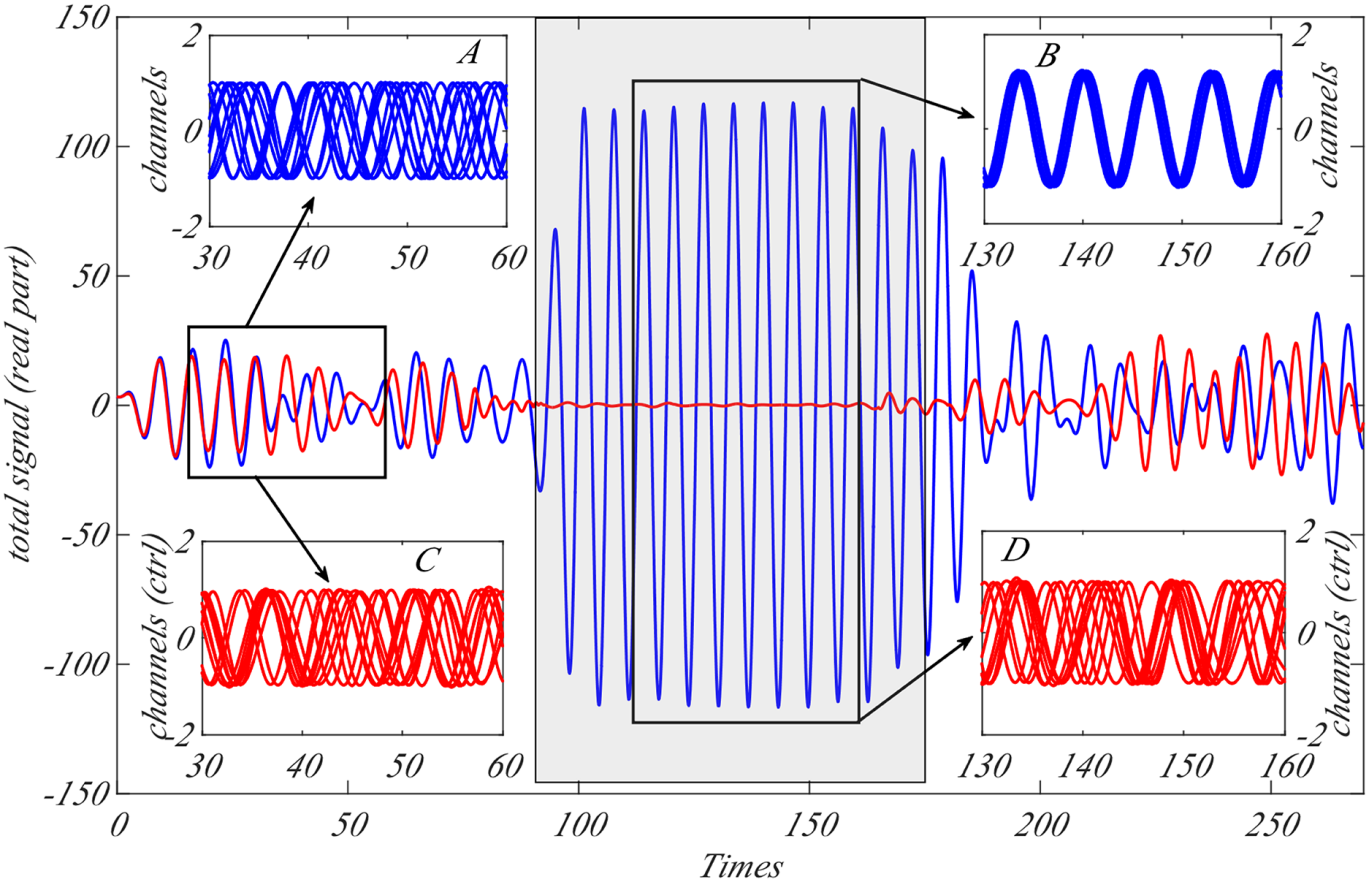
Onset of an epileptic seizure in the Stuart-Landau-like neurons population and the outcome of the controlled system. In the main plot, we represent the (real part of the) total signal ∑_*k*_ *ρ*_*k*_ cos *ϕ*_*k*_ (blue curve for the SL model and red curve for the controlled one) as a function of time for *N* = 100 coupled oscillators coupled by a Newman-Watts small-world network [55] with parameter *p* = 0.85 and *M* = 20 microelectrodes for the controlled case and *γ*/4 = 4.25. We assume *K* to be small, namely fluctuating around the average value 0.1, in the interval [0, ∼ 90]; during this period of time both systems behave similarly and do not exhibit synchronisation (see inset A for the SL model and inset C for the controlled SL model). Then we assume the coupling parameter to start to increase to eventually remain quite large, on average 0.5, in the time interval [∼ 90, ∼170]; we can observe that the SL synchronises (see inset B) while the controlled version still exhibit a non-synchronised regime (see inset D). Once the coupling parameter decreases and fluctuates again around a small value, 0.1, both systems recover the same non-synchronised state (data not shown).

As before, we performed simulations to mimic the onset of an epileptic seizure to prove the effectiveness of the control strategy applied to the Stuart-Landau model. More precisely we consider a system of *N* = 100 neurons described by the Stuart-Landau model (1) and its controlled version (15) (*M* = 20 microelectrodes and *γ*/4 = 4.25) using again a Newman-Watts network. Initially the coupling parameter fluctuates around a small value and then becomes larger. In Fig 6 we represent the total (real part of the) signal, ∑_*k*_ ℜ*z*_*k*_ = ∑_*k*_ *ρ*_*k*_ cos *ϕ*_*k*_, for the original Stuart-Landau model (blue curve) and the controlled model (red curve). In the interval [0, ∼ 90], *K* is small and neither system synchronises, as can be seen in the insets A (real part of the signal for 10 generic neurons for the original Stuart-Landau model) and C (real part of the signal for 10 generic neurons for the controlled Stuart-Landau model), the amplitude of the total signal is thus quite small. On the other hand for larger times, [∼ 90, ∼170] (roughly corresponding to the shaded central rectangular part of the figure), *K* assumes larger values than in the previous period and the Stuart-Landau system enters in a synchronised state (see inset B where we plot the real part of the signal for the same 10 generic neuronal patches of inset A) while the controlled system remains in a non-synchronised state (see inset D where again we plot the real part of the signal for the same 10 generic neurons of inset C). This corresponds to quite a large amplitude for the total signal because now the amplitudes of each single signal add coherently together.

## Discussion

In this paper, we presented a new method to control abnormal synchronisation of neuronal activity based on the Hamiltonian control formalism applied to the paradigmatic Kuramoto model. We focus on the phase dynamics which prepares the foundation for most of the basic functioning of the brain regions. As it is well-known when the coupling strength *K* exceeds a critical value, the phases of the electrical currents of the neurons of interest get locked and, due to a resonance effect, the neural signal amplifies directly affecting the behaviour. However, sometimes this behaviour is not the desirable and can be associated to neurological diseases, as in the case of epileptic seizures. Often drugs are not sufficient to control, i.e. reduce, the strength of the seizures and invasive brain stimulation becomes necessary. We therefore propose an efficient and minimally invasive control technique aimed to prevent the phase-locking and thus applicable to all cases where over-synchronisation is responsible for undesired negative effects.

Starting from a theoretical result [35], we further develop the control term and adapt it towards potential realistic applications where an abnormal synchronisation state is present, including complex (weighted) network topologies. The main idea is to effectively control the interested neuronal patches and brain regions while reducing side effects as much as possible. In terms of control strategy, this amounts to have as few microelectrodes implanted as possible, and which signal injection is directly regulated by the magnitude of the order parameter. The control term then becomes active only when needed. The method is very promising and the desynchronisation level achieved is very good compared with the standard represented by the Proportional-Differential Feedback control.

Starting from control scheme developed for the KM we are able to define a control strategy acting directly on the Stuart-Landau model widely used to describe the interaction of coupled neuronal patches [42] and numerically show its effectiveness in suppressing the synchronised state and thus the neuronal disease. The latter result is in our opinion a proof-of-concept that the presented method could be applied to deal with real cases.

The control strategy presented in this paper is purely theoretical and need further validation before envisaging a clinical implementation. First we need to carry further *in silico* investigations on more realistic topologies. The human connectome would be used for the large scale interaction between brain regions, each modelled by smaller network of interacting neural patches, would allow for an extensive investigation of how the control of neural patches within brain regions reverberates to large scale brain dynamics. This goes along the current research lines where brain regions activities are modelled using Stuart-Landau systems, whose bifurcations parameters are used to reproduce the disease we are interested in [42, 56]. The main difference with respect to the model presented here, based on a 100-nodes networks of coupled Stuart-Landau systems, is the size and the topology of the network and the possibility to have negative bifurcation parameters. However, based on our positive results (see Fig 6) and on the potential robustness of the strategy with respect to changes in the connectivity (see Fig. B in S1 Text), we are confident that this generalisation can be achieved. This first phase will also be used to precisely benchmark the goodness of the synchronisation controllability versus the invasiveness of the strategy, namely the number of used microelectrodes (*M*) and the strength of the signal (*γ*) and thus yield results directly comparable with current implementation. Parallel to these *in silico* experiments, *in vitro* experiments could be designed to test our control framework. Indeed Shew and colleagues [57] have performed experiments altering the balance between inhibition and excitation on cortical slices, and this set up could in principle be used to directly test the potential of our control framework to restore the inhibition/excitation balance. Finally, we must point out that our method provides a theoretical framework for empirically determined control strategies proposed in the literature [22–24], adding credibility to its applicability in real conditions.

## Supporting information

S1 Text Generalisation, role of topology and effectiveness of the control method. In the Supplementary Text we have discussed and proposed a generalisation of our control method to deal with weighted networks and also tested its effectiveness in network models with different topology. Furthermore, we have compared our technique with another well-known one already experimented in Parkinsonian patients, the Proportional-Differential Feedback (PDF) method. In all the cases our approach results better than the PDF method and is relatively independent on the network topology.
(PDF)
