# Supplementary material for: A minimally invasive neurostimulation method for controlling abnormal synchronisation in the neuronal activity
Source: PLoS Comput Biol. 2018 Jul 19;14(7):e1006296. doi: 10.1371/journal.pcbi.1006296 (PMC6067766; doi:10.1371/journal.pcbi.1006296)
Supplement: S1 Text — In the Supplementary Text we have discussed and proposed a generalisation of our control method to deal with weighted networks and also tested its effectiveness in network models with different topology. Furthermore, we have compared our technique with another well-known one already experimented in Parkinsonian patients, the Proportional-Differential Feedback (PDF) method. In all the cases our approach results better than the PDF method and is relatively independent on the network topology. (PDF) [file pcbi.1006296.s001.pdf]

Generalisation, role of topology and effectiveness of the control method

Malbor Asllani[1*], Paul Expert[2,3], Timoteo Carletti[1],

1 naXys, Namur Institute for Complex Systems, University of Namur, rempart de la Vierge 8, B 5000 Namur, Belgium
2 Department of Mathematics, Imperial College London, London, SW7 2AZ, UK
3 EPSRC Centre for Mathematics of Precision Healthcare, Imperial College London, London, SW7 2AZ, UK

* malbor.asllani@unamur.edu

CONTROL THE KURAMOTO MODEL DEFINED ON WEIGHTED NETWORKS

The control strategy proposed in [1] and used in the main text to desynchronise a system of coupled Kuramoto oscillators has been developed under the assumption of a binary network of interactions. Namely either two oscillators influence each other dynamics directly or they don't, and the strength of the interaction is completely captured by the parameter K. However, it can happen that in some applications the interaction strengths are heterogeneously distributed; this is in particular true in the case of the brain, where one can find "fast" and "slow" pathways determining thus a small-world functional structure, cerebral zones are close or distant one, once measured in terms of time needed for the information to travel [2, 3]. For this reason, we hereby present a straightforward extension of the model that also cover this situation.

Let us thus consider the Kuramoto model defined on a weighted symmetric network, i.e. the coupling among angles ϕ_j and ϕ_i is no longer a binary value 0 or 1 but a positive real number; one could also in principle include signed weights and/or non symmetric interactions. The model can thus be described by

$$\dot{\phi}_i = \omega_i + \sum_{j=1}^{N} W_{ij} \sin(\phi_j - \phi_i)\,, \tag{1}$$

where $W_{ij} = W_{ji} > 0$ is the weighted adjacency matrix. The original Kuramoto model is hence recovered for the choice $W_{ij} = K/N$ for all i and j, where K is the strength of the non-linearity and N - the number of oscillators - a normalising factor to make the coupling intensive.

In the case under scrutiny there exist several ways to normalise the interaction term [4], they mainly differ when we are interested in comparing networks with different sizes or considering the behaviour of the system in the thermodynamics limit, $N \to \infty$. For the scope of this work it is enough to normalise the intensity of the interaction term by using the node degree, $k_i = \sum_j A_{ij}$, where A_{ij} is the binary adjacency matrix, i.e. $A_{ij} = 1$ if and only if $W_{ij} \neq 0$. The model can thus be rewritten as

$$\dot{\phi}_i = \omega_i + \sum_{j=1}^{N} \frac{W_{ij}}{k_i} \sin(\phi_j - \phi_i)\,. \tag{2}$$

To go one step forward let us define an order parameter, as we did for the Kuramoto model, allowing to rewrite the system in a self-contained way using a mean-field approach. We observe however that in the present case we must use a local order parameter, depending of the node index i:

$$R_i^{(w)} e^{\iota\psi_i} := \sum_{j=1}^{N} \frac{W_{ij}}{k_i} e^{\iota\phi_j}\,, \tag{3}$$

from which one can straightforwardly obtain

$$R_i^{(w)} \sin(\psi_i - \phi_i) = \sum_{j=1}^{N} \frac{W_{ij}}{k_i} \sin(\phi_j - \phi_i)\,, \tag{4}$$

and thus rewrites Eq. (2) as

$$\dot{\phi}_i = \omega_i + R_i^{(w)} \sin(\psi_i - \phi_i)\,. \tag{5}$$

The next step consists in observing that the equation above can still be obtained from a Hamiltonian formalism (see Eqs.(5) and (6) in the main text where $A_{ij} \to W_{ij}/k_i$) and thus one can build a control term in a very similar way. A long but straightforward computation eventually provides the following control term:

$$\begin{aligned} h_i^{(w)} \quad & (\phi_1, \dots, \phi_N) = \\ -\tfrac{1}{4} \quad & \Big[\sum_j \frac{W_{ij}}{k_i} \cos(\phi_j - \phi_i) \sum_l \frac{W_{il}}{k_i} \frac{1}{\omega_l - \omega_i} \cos(\phi_l - \phi_i) + \sum_j \frac{W_{ij}}{k_i} \frac{1}{\omega_j - \omega_i} \sin(\phi_j - \phi_i) \sum_l \frac{W_{il}}{k_i} \sin(\phi_l - \phi_i) + \\ - \quad & \sum_l \left(\frac{W_{il}}{k_i} \cos(\phi_i - \phi_l) \sum_j \frac{W_{jl}}{k_j} \frac{1}{\omega_j - \omega_l} \cos(\phi_j - \phi_l) + \frac{W_{il}}{k_i} \frac{1}{\omega_i - \omega_l} \sin(\phi_i - \phi_l) \sum_j \frac{W_{jl}}{k_j} \sin(\phi_j - \phi_l) \right) \Big] , \end{aligned} \tag{6}$$

which can be further simplified after the introduction of

$$\tilde{R}_i^{(w)} e^{\iota \Psi_i} := \sum_{j=1}^{N} \frac{W_{ij}}{k_i} \frac{e^{\iota \phi_j}}{\omega_j - \omega_i} . \tag{7}$$

We thus eventually get

$$h_i^{(w)}(\phi_1, \dots, \phi_N) = -\frac{1}{4} \left[R_i^{(w)} \tilde{R}_i^{(w)} \cos(\psi_i - \Psi_i) - \mathcal{B}_i^{(w)} \right] , \tag{8}$$

where $\mathcal{B}_i^{(w)}$ has been defined in the last equality.

The control term (8) can be further simplified to make it operational along the same ideas developed in the main text, namely by replacing the phase Ψ_i with the angle ϕ_i, and neglect the term $\mathcal{B}_i^{(w)}$. We also take into account the presence of an electromagnetic field potential [5, 6] created by the controlled microelectrodes acting on the neighbouring neurons. In conclusion, we introduce a new stimulation signal, $S_i^{stim,w}$, generated on the position of the i-th neuron by the potential produced by the microelectrodes located in all the controlled neuronal patches:

$$S_i^{stim,w}(\phi_1, \dots, \phi_M) = c_s \sum_{l=1}^{M} e^{-2r_{il}} \hat{h}_l^{(w)}(\phi_1, \dots, \phi_M) ; \tag{9}$$

where r_{il} and c_s are respectively the distance of node i from the origin of the electromagnetic field l, and the strength of the potential which in our case is taken to be $c_s = 1$, finally $M \ll N$ is the number of directly controlled nodes. The proposed control strategy taking into account the weights is thus:

$$\dot{\phi}_i = \omega_i + R^{(w)} \sin(\Psi_i - \phi_i) + S_i^{stim,w}(\phi_1, \dots, \phi_M) . \tag{10}$$

SOME MODELS OF WEIGHTED NETWORKS

The proposed control strategy is independent from the network topology considered and the weights distribution. However for a sake of completeness and for the relevance with the proposed application, we hereby consider three classes of weighted networks: an all-to-all connectivity topology, an Erdős-Rényi network [7] and a Newman-Watts [8] coupling. In all the cases the weights are uniformly randomly distributed.

The results reported in the following Fig. 1SM show that the improved control method is able to achieve a strong degree of desynchronisation for non-linear oscillators evolving according to the Kuramoto model. We then define the (de)synchronisation level of the system using the Kuramoto order parameter $R(t) = |\sum_j e^{\iota \phi_j(t)}|/N$. All the considered networks are made by $N = 100$ nodes, $M = 20$ microelectrodes are used to measure and to control the whole system and the control parameter has been fixed to $\gamma/4 = 20$. The natural frequencies are drawn from the normal distribution $\mathcal{N}(1, 0.01)$, and the initial angles are uniformly randomly drawn in $[0, 2\pi]$. In the left panel we report the case of a complete topology where all nodes are connected with all other nodes and the weights are drawn from an uniform distribution $\mathcal{U}[0.7, 0.9]$, and then normalised by the node degree $W_{ij} \mapsto W_{ij}/k_i$. One can observe that the original Kuramoto model exhibits a strong synchronisation with the order parameter R very close to one (blue curve), on the other hand the controlled model (red curve) shows a very small R, thus denoting a high level of desynchronisation. In the middle panel, we consider as underlying topology an Erdős-Rényi network with parameter $p = 0.8$, the probability that a link exists between two nodes; existing links have their weights drawn from the same uniform distribution used

above. One can again observe that after a very short transient phase during which the controlled system exhibits an order parameter of the same order of the original Kuramoto model, the controlled system desynchronises (red curve) while the original Kuramoto model does the opposite (blue curve). Finally, in the right panel, we present the results concerning the Newman-Watts network with parameter $p = 0.85$ and the conclusions are similar to the previous ones, the controlled system (red curve) is strongly desynchronised while the original model doesn't.

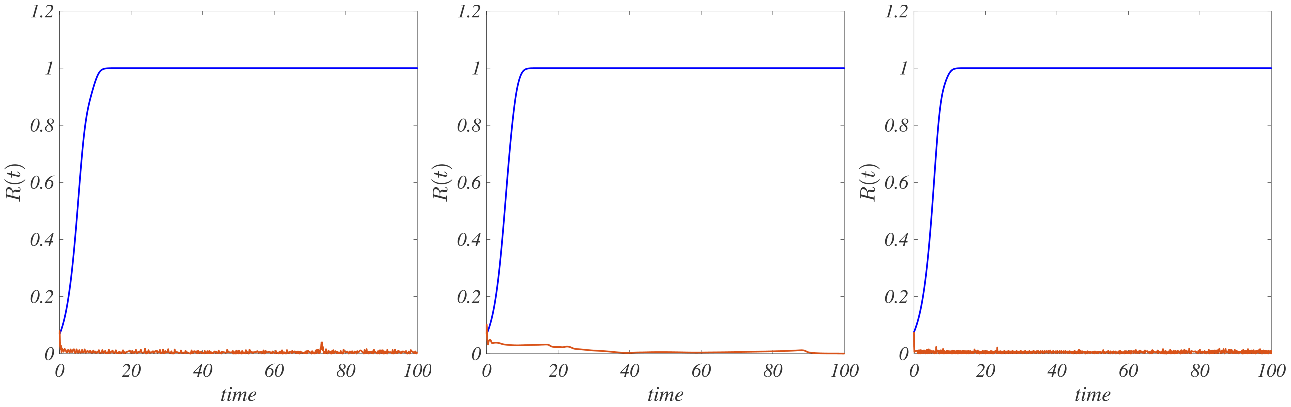

FIG. 1: Control of weighted Kuramoto model. Order parameter, $R(t) = |\sum_j e^{\iota\phi_j(t)}|/N$, vs time. The weights are uniformly random distributed, $W_{ij} \in \mathcal{U}[K-d, K+d]$, $K = 0.8$ and $d = 0.1$, while the underlying topologies change. All-to-all coupling (left panel), Erdős-Rényi with parameter $p = 0.8$ (middle panel) and Newman-Watts with parameter $p = 0.85$ (right panel). In all the presented cases the networks are made by $N = 100$ nodes, weights are normalised by the node degree $W_{ij} \mapsto W_{ij}/k_i$, the natural frequencies are drawn from the distribution $\mathcal{N}(1,\sigma)$, $\sigma = 0.01$ and the initial angles uniformly randomly drawn in $[0, 2\pi]$. The control term uses $M = 20$ nodes and its strength is given by $\gamma/4 = 20$.

THE ROLE OF THE NETWORK TOPOLOGY IN THE DESYNCHRONISATION ISSUE

In the main text, we presented the result concerning the impact of the control parameters M and γ to achieve the desynchronised state, under the assumption of an all-to-all unweighted topology (Fig. 2 main text, and here reported in panel (d) of Fig. 2SM to help the comparison). The outcome is that a high level of desynchronisation (dark blue) can be achieved using few microelectrodes, i.e. take small values for M, provided a strong enough control is used, namely assume a large enough value for γ. The opposite is also true, one can use a small coupling γ but then more mictroelectrodes are needed. In the remaining panels of Fig. 2SM, we analyse the same question assuming a Newman-Watts topology with binary weights and we study the impact of the parameter p responsible for the creation of shortcuts. Once p is small, the network is close to a $1D$-regular lattice (with coordination number $k = 6$ in the present case), i.e. very few new links are added; for larger p the network becomes more dense and in the limit $p = 1$ we recover a complete network. For any fixed parameters values $(M, \gamma/4)$, we report the asymptotic value of the Kuramoto order parameter $R = |\sum_j e^{\iota\phi_j}|/N$ averaged over 25 independent realisation. We repeated this construction for three Newman-Watts networks, with a high link density ($p = 0.85$, panel c), with a medium link density ($p = 0.5$, panel b) and a diluted link density ($p = 0.25$ panel a). The following colour coding has been used: the dark (blue) corresponds to small values of $\langle R\rangle$ meaning thus a desynchronised state, while the light (yellow) is associated with large value for the average order parameter, i.e. synchronisation. Taking into account that the all-to-all network of Fig. 2 (main text) and panel d of Fig 2SM is the limit of a Newman-Watts network with parameter $p = 1$, our results confirm the intuition that the smaller the M the larger should be the strength to achieve a desynchronised state; however we can also emphasise the fact that diluted networks are more easily desynchronised than denser ones, the dark (blue) zone in panel a is much larger than in the remaining panels. Finally, we observe that for small p the system almost never synchronises (no light yellow spots) for all the used values of $(M, \gamma/4)$; indeed in the case of a regular lattice corresponding to $p = 0$ the system never synchronises (data not shown).

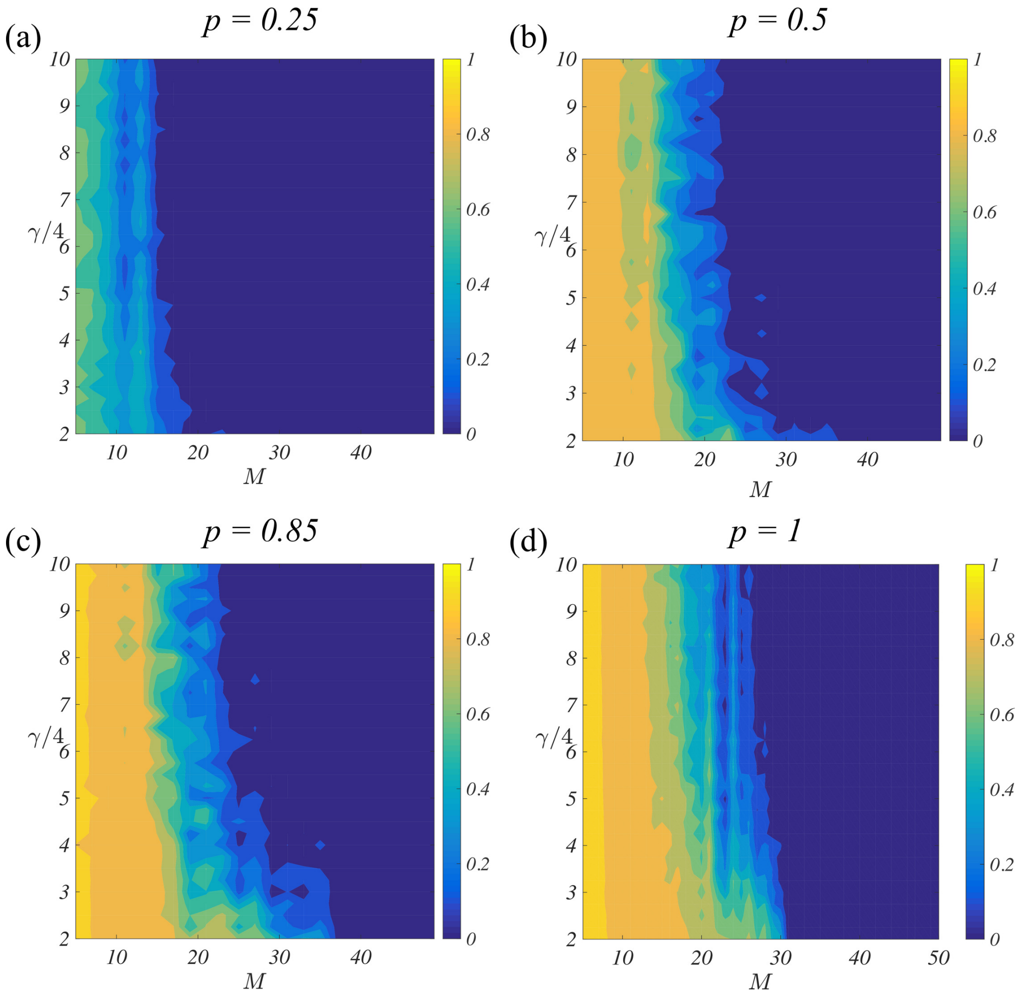

FIG. 2: The parameters M and γ and the desynchronisation achievement for a Newman-Watts network. The Kuramoto order parameter $\langle R(t)\rangle$, averaged over 25 independent replicas, is shown as a function of number of microelectrodes (M) and the strength of the control ($\gamma/4$). For each couple $(M, \gamma/4) \in [5, 50] \times [2, 10]$ we numerically simulate the controlled system on a Newman-Watts network made by $N = 100$ oscillators and parameter $p = 0.25$ (panel a), $p = 0.5$ (panel b), $p = 0.85$ (panel c) and all-to-all coupling, i.e. $p = 1$ (panel d). The coupling parameter is $K = 0.5$ and the natural frequencies are drawn from a normal distribution $\mathcal{N}(1, \sigma)$, $\sigma = 0.01$ and the initial angles uniformly randomly drawn in $[0, 2\pi]$.

COMPARISON WITH THE PROPORTIONAL-DIFFERENTIAL FEEDBACK (PDF) CONTROL TECHNIQUE

The aim of this section is to compare the control method proposed here (for unweighted and complete networks) with the proportional-differential feedback (PDF) control method [9], whose importance has been already proved in the literature.

Let us start by briefly recalling the PDF method and fix, for the sake of completeness, the Kuramoto model on binary complete networks as working problem. In this way we can easily compare the method with the one presented in the main text. The method relies on the hypothesis of dividing the population of oscillators into two groups, the first one made by N_1 oscillators whose mean-field signal will be measured during the time evolution and then used to directly control the second group of N_2 oscillators. This translates into the following equations for the oscillators

phases ϕ_j:

$$\begin{aligned}\dot{\phi}_j &= \omega_j + \frac{K}{N}\sum_{l=1}^{N}\sin(\phi_l-\phi_j) - \Theta(j-N_1)F_j \\ F_j &= \frac{P}{N_1}\sum_{l=1}^{N_1}\sin(\phi_l-\phi_j) + \frac{D}{N_1}\sum_{l=1}^{N_1}\dot{\phi}_l\cos(\phi_l-\phi_j)\,,\end{aligned} \tag{11}$$

where $\Theta(\cdot)$ is the Heaviside function, $\Theta(k)=0$ if $k\leq 0$ and $\Theta(k)=1$ if $k\geq 1$, its role being thus to limit the feedback action only to nodes whose index satisfies $j\geq N_1+1$, i.e. nodes of the second group. The control signal is composed by two terms, the leftmost one being *proportional* to the mean-field signal of the first group of oscillators, being P the proportionality constant. The rightmost term contains the time *derivative* of the mean-field signal of the first N_1 oscillators, namely it is the average of the derivative of the phases of the first group modulated with a signal retarder by a fourth of the period, i.e. involving the cosine function instead of the sine one. D is a parameter defining the strength of this second term. Let us observe that this empirically based functional dependence, (cos), is consistent with the control strategy we derived based on the Hamiltonian control theory, which thus can support the goodness of this choice.

Having fixed the strength of the non-linear term in the Kuramoto model, K, (and of course the number N of oscillators) the PD controlled systems (11) depends on three free parameters: N_2 the number of controlled oscillators, P the strength of the proportional term and D the strength of the differential term. Let us observe a main difference with respect to our scheme; if one uses few controllers in the PDF strategy, i.e. N_2 is small, then automatically one assumes to be able to acquire the signal from a large number of neurons, namely $N_1=N-N_2$ is large, this reflects the distinction between measuring nodes and controlled nodes and can be an issue in real applications. This division does not exist in our setting, where the same microelectrodes are used to acquire the signal and then to re-inject the controlled signal; in this way we can use a small number of controllers without the need to measure the signal from a large portion of the system. In other words, our model has more local flavour.

One can show [9] that the PD control algorithm involving both the proportional and differential feedback components is more efficient than the case $D=0$, moreover one can compute [9] the desynchronisation thresholds, $\tilde{P}$ and $\tilde{D}$, to achieve a desynchronised regime and prove that the latter increase if the number of oscillators in the second group, N_2, decreases. One needs larger parameters values for P and D to destabilise the synchronous state once N_2 is small. This behaviour is similar to the one of our model.

COMPARE THE TWO CONTROL STRATEGIES.

The goal of this section is to compare the PDF control method and the one we proposed in the main text based on the Hamiltonian control theory. Let us recall that the PD-control is based on a division of the oscillators ensemble into two groups, the first one made by N_1 oscillators whose signal will be measured and the second one composed by $N_2=N-N-1$ oscillators upon which the control will directly act. As already observed our model select M oscillators among N whose signal is measured and the same M oscillators are used to control the system. So to compare the two methods we can assume that $N_2=M$, this value being a proxy for the invasiveness of the method. A second proxy for the invasiveness, is the strength of the injected signal; in our scheme this is given by $\gamma/4$ (see Eq. (12) in the main text) while in the PD control one can assume this strength given by $P+D$, being the control signal the sum of bounded functions whose maximal amplitude are P and D.

The results of the comparisons are reported in Fig. 3SM where we show the action of the two control strategies for several values of the strength parameters, P, D and $\gamma/4$, and number of controlled nodes, $N_2=M$, for $N=100$ oscillators coupled using an all-to-all setting. The Kuramoto parameter has been set to $K=0.5$ and the natural frequencies drawn from the distribution $\mathcal{N}(1,0.01)$, in such a way the uncontrolled system does synchronise (blue line in the panels). In the left panel we report the case of a mild control, $P=D=10$, hence $\gamma/4=20$, using few controlled oscillators, $N_2=M=20$; we can observe that the PD-control reduces the synchronisation by a factor 5 while our method is able to completely set the system into a strongly desynchronised state. The same result is achieved by both control schemes once the strength is increased (middle panel), $P=D=20$ hence $\gamma/4=40$. Using again the mild control strength but increasing the number of controlled nodes ($N_2=M=50$, right panel) one can achieve again a strongly desynchronised state using both strategies. Such results support our claim that the control strategy hereby proposed is minimally invasive both in terms of number of microelectrodes needed and strength of the signal used to achieve a desynchronisation state.

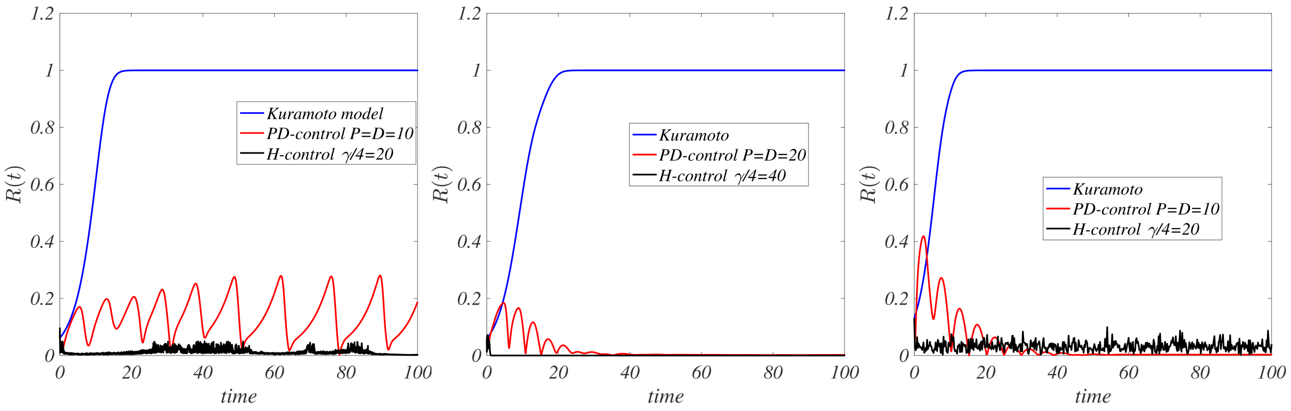

FIG. 3: Comparison of the two control strategies. We plot the order parameter $R(t) = |\sum_j e^{\iota\phi_j(t)}|/N$ as a function of time for the original Kuramoto model (blue line), the PD-controlled one (red curve) and our control method based on the Hamiltonian control (black line), for several values of the controls parameters, P, D and $\gamma/4$, and number of controlled oscillators, $N_2 = M$. Left panel: $N_2 = M = 20$, $P = D = 10$ and $\gamma/4 = 20$; middle panel: $N_2 = M = 20$, $P = D = 20$ and $\gamma/4 = 40$; right panel: $N_2 = M = 50$, $P = D = 10$ and $\gamma/4 = 20$. The coupling parameter is fixed to $K = 0.5$, the natural frequencies are drawn from the distribution $\mathcal{N}(1, \sigma)$, $\sigma = 0.01$ and the initial angles uniformly randomly drawn in $[0, 2\pi]$.

To gain a more global view of the PDF control scheme an compare it with our method, we perform a study of the impact of the parameters N_2, P and D on the desynchronisation issue for the Kuramoto model (on a complete unweighted network). Using several combinations of the involved parameters, we realised independent simulations of the PDF control system and we measure the averaged (over the realisations) order parameter $\langle R \rangle$. The results are reported in Fig. 4SM, where we varied P and N_2, while D is fixed and equal to $D = 0$ (left panel) and to $D = 10$ (right panel). One observe that the latter control strategy gives the best results in terms of desynchronisation, the zone associated with a small $\langle R \rangle$ (green-blue) being larger. Such results should be compared with the ones reported in Fig. 2 (main text) or panel (d) of Fig. 2, where a similar analysis has been done using our control scheme. In particular the parameters used in the left panel of Fig. 4 corresponds to panel (d) of Fig. 2, $\gamma/4 = P + D = P$ ranges in $[2, 10]$ (being $D = 0$) and $N_2 = M$ range in $[5, 50]$. While the behaviour is globally the same, the use of the same colour code easily allows to appreciate the fact that in panel (d) of Fig. 2 the desynchronised state is stronger, namely $\langle R \rangle$ smaller (darker blue). Moreover (see right panel), at fixed $N_2 = M$, the PD-control requires a larger control strength (observing the vertical scale and the choice $D = 10$, we conclude that the strength varies in $[12, 50]$) with respect to our method, to achieve an equivalent level of desynchronisation.

[1] Gjata, O., Asllani, M., Barletti, L. and Carletti, T. Using Hamiltonian control to desynchronize Kuramoto oscillators. *Phys. Rev. E* **95**(2), 022209 (2017).
[2] P. Expert, R. Lambiotte, D. R. Chialvo, K. Christensen, H. J. Jensen, D. J. Sharp, and F. E. Turkheimer, Journal of the Royal Society Interface **8**, 472 (2011).
[3] F. E. Turkheimer, R. Leech, P. Expert, L.-D. Lord, and A. C. Vernon, Neuroscience and Biobehavioral Reviews **55**, 211 (2015).
[4] Alex Arenas and Albert Díaz-Guilera and Jurgen Kurths and Yamir Moreno and Changsong Zhou, *Synchronization in complex networks*, Physics Reports, **469** (3), (2008), pp. 93 - 153
[5] Nunez, P. *Electrical fields of the brain*, Oxford University Press (1981).
[6] Rinzel, J. and Ermentrout, G. Analysis of the neural excitability and oscillations, in *Methods in Neuronal modelling from synapses to networks* (eds. Koch, C. and Segev, I.) 135–169 MIT Press (1989).
[7] P. Erdős and A. Rényi, *On the evolution of random graphs*, Publ. Math. Inst. Hung. Acad. Sci., **5**, (1960), pp.17 - 61.
[8] Newman, M. E. J. and Watts, D. J. Scaling and percolation in the small-world network model. *Phys. Rev. E*, **60**, 7332 (1999).
[9] K. Pyragas, O. V. Popovych and P. A. Tass, *Controlling synchrony in oscillatory networks with a separate stimulation-registration setup*, Euro. Phys. Lett., **80**, (2007), pp. 40002-1–6

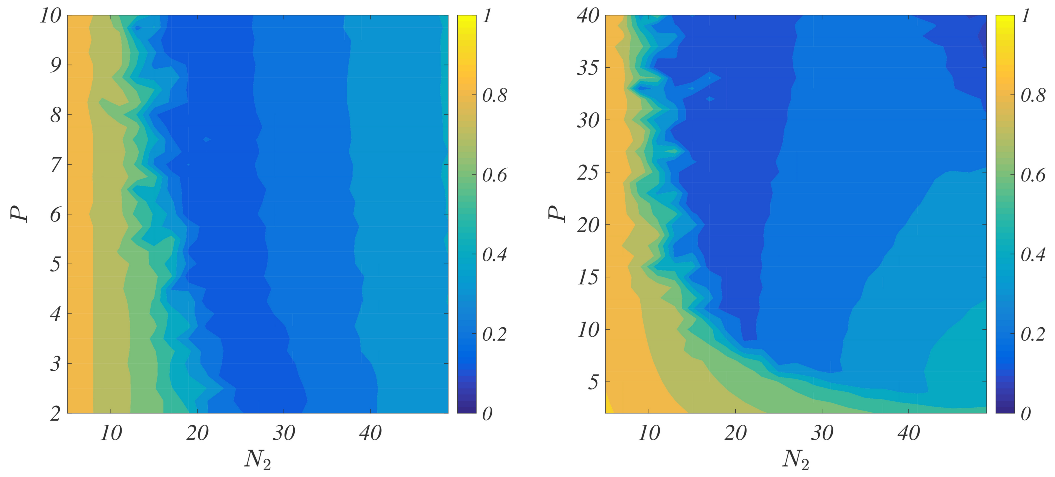

FIG. 4: The role of N_2, P and D on the desynchronisation for the PD-control. The average Kuramoto order parameter $\langle R(t)\langle$ is reported as a function of number of controlled oscillators, N_2, and the control strength, P, for several values of D ($D = 0$ left panel and $D = 10$ right panel). For each couple $(N_2, P) \in [5, 50] \times [2, 10]$ (left panel) and $(N_2, P) \in [5, 50] \times [2, 40]$ (the right panel), we numerically simulate the PD-controlled system on a ensemble of $N = 100$ oscillators coupled using an all-to-all scheme; each point being the average of 25 independent realisations. The coupling parameter is $K = 0.5$ and the natural frequencies are drawn from a normal distribution $\mathcal{N}(1, \sigma)$, $\sigma = 0.01$ and the initial angles uniformly randomly drawn in $[0, 2\pi]$.
